# High-throughput chinmedomics-based prediction of effective components and targets from herbal medicine AS1350

**DOI:** 10.1038/srep38437

**Published:** 2016-12-02

**Authors:** Qi Liu, Aihua Zhang, Liang Wang, Guangli Yan, Hongwei Zhao, Hui Sun, Shiyu Zou, Jinwei Han, Chung Wah Ma, Ling Kong, Xiaohang Zhou, Yang Nan, Xijun Wang

**Affiliations:** 1 Sino-America Chinmedomics Technology Cooperation Center, Chinmedomics Research Center of TCM State Administration, National TCM Key Laboratory of Serum Pharmacochemistry, Laboratory of Metabolomics, Heilongjiang University of Chinese Medicine, Heping Road 24, Harbin 150040, China; 2 Infinitus (China) Company Ltd, Guangdong Province, China; 3 Department of Pharmaceutical Analysis, Heilongjiang University of Chinese Medicine, Heping Road 24, Harbin 150040, China; 4 State Key Laboratory of Quality Research in Chinese Medicine, Macau University of Science and Technology, Avenida Wai Long, Taipa Macau

## Abstract

This work was designed to explore the effective components and targets of herbal medicine AS1350 and its effect on “Kidney-Yang Deficiency Syndrome” (KYDS) based on a chinmedomics strategy which is capable of directly discovering and predicting the effective components, and potential targets, of herbal medicine. Serum samples were analysed by UPLC-MS combined with pattern recognition analysis to identify the biomarkers related to the therapeutic effects. Interestingly, the effectiveness of AS1350 against KYDS was proved by the chinmedomics method and regulated the biomarkers and targeting of metabolic disorders. Some 48 marker metabolites associated with alpha-linolenic acid metabolism, fatty acid metabolism, sphingolipids metabolism, phospholipid metabolism, steroid hormone biosynthesis, and amino acid metabolism were identified. The correlation coefficient between the constituents *in vivo* and the changes of marker metabolites were calculated by PCMS software and the potential effective constituents of AS1350 were also confirmed. By using chinmedomics technology, the components in AS1350 protecting against KYDS by re-balancing metabolic disorders of fatty acid metabolism, lipid metabolism, steroid hormone biosynthesis, *etc*. were deduced. These data indicated that the phenotypic characterisations of AS1350 altering the metabolic signatures of KYDS were multi-component, multi-pathway, multi-target, and overall regulation in nature.

The 2015 Nobel Prize in Physiology or Medicine was won by a pharmacologist, Youyou Tu, who was the first China-based Nobel scientist. Tu’s discovery of artemisinin, a key antimalarial drug rooted in ancient Chinese herbal medicine, promoting traditional Chinese medicine (TCM) to the forefront among global research communities. Herbal medicine, such as TCM, has been extensively applied in preventing diseases or safe guarding health in oriental countries for thousands of years and it has received widespread recognition because of its reliable therapeutic effects^1^. Due to the development of technologies and the effectiveness of evaluation methods used, metabolomics technology and serum pharmaco-chemistry are beneficial to the assessment of the characteristics of TCM^2^,^3^. However, because of the complexity of the herbs used, the identification of effective constituents *in vivo* related to the efficacy mechanisms has formed a bottleneck in the understanding and data flow^4^. It is imperative for modern medical scientists to discover a biological language elucidating the efficacy of TCM scientifically. Under this background, chinmedomics has been proposed by our team^5^. It is a newly defined theory and research method for expressing the efficacy of TCMs based on the biomarkers’ discovery of syndromes and elucidation of the efficacy of TCM formulae. It will shed new lights on the discovery of effective constituents, and finally clarify the scientific value of TCM.

The efficacy of TCM is directly related to syndrome and TCM formulae, however, the vagueness of syndromes and the complexity of formulae make the diagnosis and evaluation of the efficacy of TCMs difficult, which greatly limits the discovery of effective constituents of TCMs. Therefore, biochemical characteristics of syndromes and the efficacy of formulae are key scientific problems hindering TCM. Our team has engaged in the innovation and research methods of TCM for a long time, and aim to solve the key scientific issues such as the nature of the biochemical essence of syndrome and efficacy of TCM formulae. In the early 1990 s, we established the theory and method of serum pharmaco-chemistry of TCM, providing a methodology for the discovery of constituents *in vivo* from TCMs, but the constituents had not been linked with the efficacy of a formula due to the lack of biomarkers of the syndrome^6^. In the 21st century, we integrated serum pharmaco-chemistry of TCM with metabolomics, developed a theory and systematic method for elucidating the biochemical essence of syndrome and efficacy of TCMs as well as the effective material basis thereof, and this theory and method have been defined as chinmedomics^7^. Briefly, using metabolomic technology to clarify the biomarkers of a syndrome, we evaluate the efficacy of TCM formulae, using serum pharmaco-chemistry to discover the active constituents *in vivo* originating from TCM formulae under effective conditions, then analysed the correlation between the exogenous constituents of formulae *in vivo* and endogenous biomarkers of each syndrome, and finally find the effective constituents were highly associated with the clinical efficacy of the formulae.

AS1350 is a TCM formulae consisting of eight herbs: *Cornua Cervi Pantotrichum(Lu-Rong), Cinnamomi Cortex (Rou-Gui), Radix Rehmanniae Praeparata (Shu-Di-Huang), Schisandra Chinensis Fructus (Wu-Wei-Zi), Barbary Wolfberry Fruit (Gou-Qi-Zi), Semen Juglandis (He-Tao-Ren), Arillus Longan(Long-Yan-Rou)* and *Fructus Ziziphi Jujubae (DA-Zao)*. All of these herbs were confirmed as being able to regulate the neuroendocrine hypofunction^8^,^9^,^10^,^11^,^12^,^13^,^14^,^15^, which is closely related to multiple disordered metabolic pathways of “Kidney-Yang deficiency syndrome” (KYDS) such as damage to the hypothalamic-pituitary-target gland (adrenal, thyroid, and gonad) axis^16^,^17^,^18^,^19^,^20^. However, the therapeutic mechanism, effective constituents, and potential targets of AS1350 effects on KYDS are unclear. In this study, ultra-high performance liquid chromatography combined with triple TOF mass spectrometry (UPLC-TOF/MS/MS) was employed to detect the global profile of endogenous metabolites and exogenous constituents from serum samples. This work was designed to explore the effective components and potential targets of AS1350 and its effects on KYDS based on a chinmedomics strategy.

## Results

### Clinical chemistry results and histological changes

**Figure 1:**
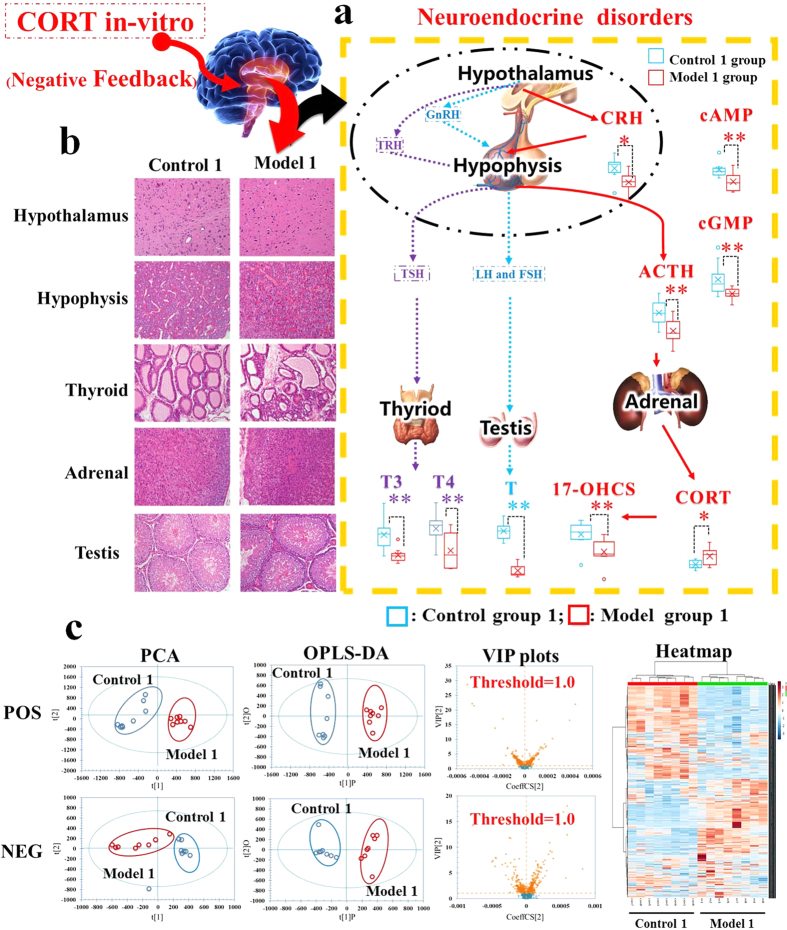
The evaluation system diagrams of Kidney-Yang Deficiency Syndrome related to neuroendocrine hypofunction (day 22). (**a**) H&E staining for histological evaluation. The pictures of KYDS model rats showed that the number of depauperate hypothalamic neurons was reduced, the counts of basophilic cells in hypophysis was decreased, the atrophic adrenocorticals were found by observing thinning of the cortex, the thyroid follicules were atrophic, deformed, and the interstitial fibrous matter around the thyroid follicules were proliferated therein. (**b**) The biochemical characteristics for evaluation of neuroendocrine system. Significant changes in the concentrations of CRH, ACTH, 17-OHCS, T3, T4, T, cAMP, and cGMP were decreased in KYDS model rats (Student’s *t*-test; *significant difference from control group 1 at *p* < 0.05, **significant difference from control group 1 at *p *< 0.01). (**c**) Multivariate data analyses resulting from the UPLC/MS spectra of serum samples. Score plots of PCA discriminated control group 1 and model group1, then score plots of VIP analysed by OPLS-DA extracted the significant contribution of different ions to the separation between control group 1 and model group1 rats. Meanwhile, a heat-map of the metabolites in two groups was derived from MetaboAnalyst 3.0 software, which exhibited a significant difference between control group 1 and model group1 rats.

On day 22, compared with control group 1, the serum CORT level was elevated significantly, whereas, the levels of CRH, ACTH, 17-OHCS, T3, T4, T, cAMP, and cGMP were decreased in model group 1 (Fig. 1a). All these results indicated that the neuroendocrine immune system of model group rats was in a state of inhibition, implying that the rat model of KYDS was established successfully. Meanwhile, pathological hypothalamic, pituitary, adrenal, and thyroid sections with H&E staining were carried out proving the extent of KYDS (Fig. 1b). The pathological histology results showed that the typical pathological features of HPA and HPT were inhibited in model group 1: the number of depauperate hypothalamic neurons was reduced and the counts of basophilic cells in hypophysis was decreased; besides, the atrophic adrenocorticals were found by observing thinning of cortex; the thyroid folliculars were atrophic and deformed; furthermore, the interstitial fibrous matter seen around thyroid follicular was proliferated.

**Figure 2:**
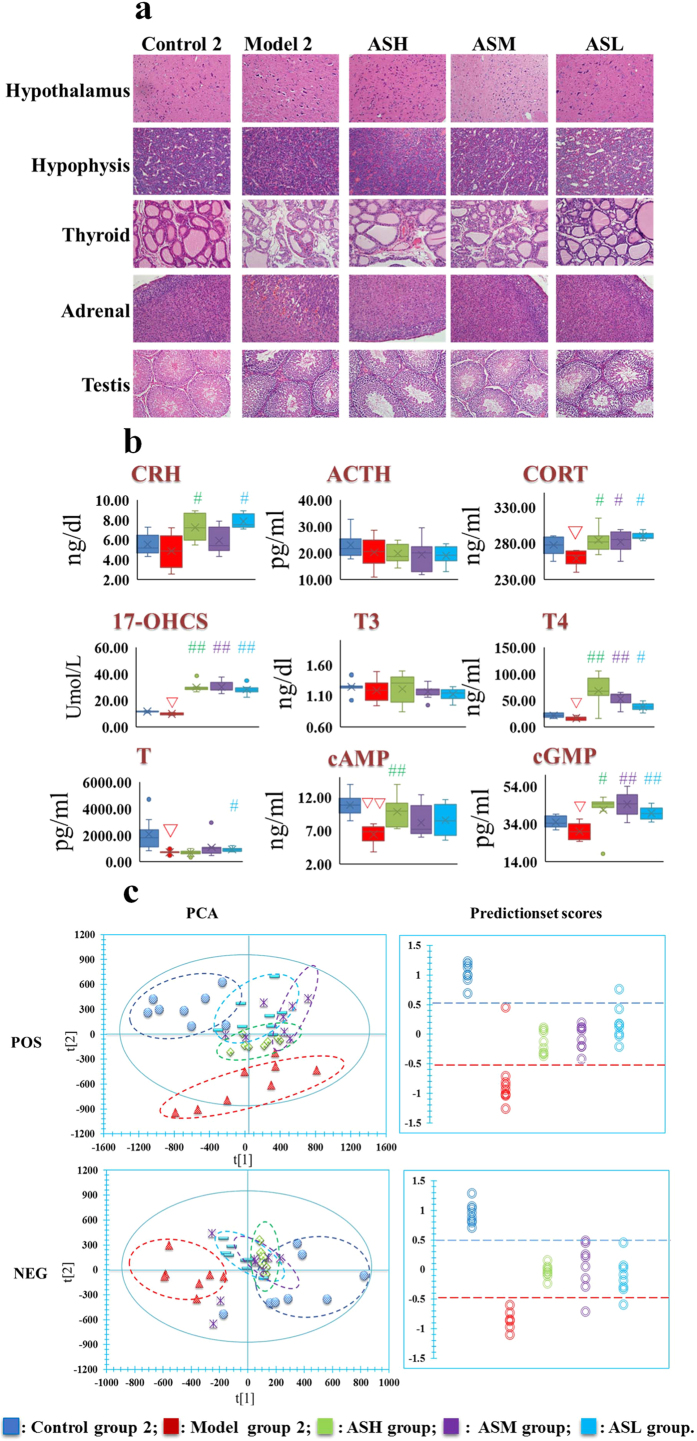
The evaluation system diagrams of the therapeutic effects of AS1350 on Kidney-Yang Deficiency Syndrome (day 43). (**a**) H&E staining results of hypothalamus, pituitary, thyroid, adrenal glands, and testes in the rats of control group 2, model group 2, and AS1350-treated group as observed by visible light microscope: all of the evidence showed that the extent of the treatment effect of AS1350 was proved by morphology results: neuronal cells in hypothalamus and basophils were increased, adrenocortical cells were arranged evenly and the atrophic cells were decreased, thyroid follicles were large and plump as well as the interstitial hyperplasia also being reduced to a regular arrangement. (**b**) The biochemical characteristics used for evaluation of the therapeutic effects of AS1350 on Kidney-Yang Deficiency Syndrome. Box and whisker diagrams comparing the level of hormones in neuroendocrine systems with each group (control group 1, model group 2, and AS1350-treated groups). After the treatment of AS1350, various levels of CORT, 17-OHCS, T, T4, cAMP, and cGMP in treatment groups returned to the level of control group 2 (1-way ANOVA with a Bonferroni correction; ∇ significant difference from control group 2 at *p *< 0.05/4, ∇∇ significant difference from control group 2 at *p *< 0.01/4; #significant difference from model group 2 at *p *< 0.05/4, ## significant difference from model group 2 at *p *< 0.01/4.). (**c**) Metabolomic *in vivo* evaluation of AS1350. PCA plots showed separation between control group 2, model group 2, and AS1350-treated. AS1350-treated groups were closer to control group 2 than model group 2, which suggested that AS1350 could reverse the pathological process of KYDS. The prediction set also annotated the therapeutic effect of each dose of AS1350, which showed the same results as the PCA plots.

Figure 2a showed that the hypothalamic neurons, adrenocortical cells and thyroid follicles were still severely atrophic, indicating that the neuroendocrine immune system of KYDS rats remained in an inhibited state after 21 days’ spontaneous recovery. On day 43, the biochemical results showed that the levels of CORT, 17-OHCS, T, T4, cGMP, and cAMP in model group 2 were significantly decreased compared with control group 2 (Fig. 2b). However, after treatment with AS1350, various levels of CORT, 17-OHCS, T, T4, cAMP, and cGMP in treatment groups could be retraced to the level of control group 2 and the extent of treatment effect was further proved by morphology results: neuronal cell counts in the hypothalamus and basophils were increased, adrenocortical cells were arranged evenly and the number of atrophic cells was decreased significantly, thyroid follicles were large and plump as well as the interstitial hyperplasia also being reduced to a regular arrangement.

### Metabolic profiling and multivariate data analysis results

Firstly, a principal component analysis (PCA) of serum profiles was performed to obtain a global overview of response in rat injected exogenous corticosterone subcutaneously and administrated to AS1350. The relevant information of PCA analysis in score plots were shown in Figs 1c and 2c: each point represented an individual sample, rats of model groups were completely separated from control groups, indicating that the metabolic networks of KYDS model rats were disordered on day 22, and the KYDS rats could not recover to control in spite of 21 days’ spontaneous recovery. However, due to the effects of AS1350, rats in treatment groups were different from model group 2 and closer to control group 2, indicating that AS1350 played an aggressive role in reversing KYDS included by exogenous corticosterone.

Then, OPLS-DA was conducted for further analysis and the feature ions were considered as potential biomarkers for KYDS. From the corresponding VIP plots in Fig. 1c, ions furthest from the origin were regarded as potential biomarkers responsible for the difference between control group 1 and model group 1. The VIP plots from OPLS-DA with a threshold of 1.0 and *p *< 0.05 (Student’s *t*-test) were generated to identify the metabolites which significantly contributed to the clustering between groups. Some 48 endogenous metabolites were identified as potential biomarkers (listed in Table S1) and the related pathways were performed by Kyoto Encyclopedia of Genes Genomes (http://www.genome.jp/kegg/), Human Metabolome Database (http://www.hmdb.ca/), and MetaboAnalyst 3.0 (http://www.metaboanalyst.ca/MetaboAnalyst/). The results suggested that fatty acid metabolism, sphingolipids metabolism, phospholipid metabolism, steroid hormone biosynthesis, and amino acid metabolism were involved in the pathological process of KYDS.

### *In vivo* components of AS1350

**Figure 3:**
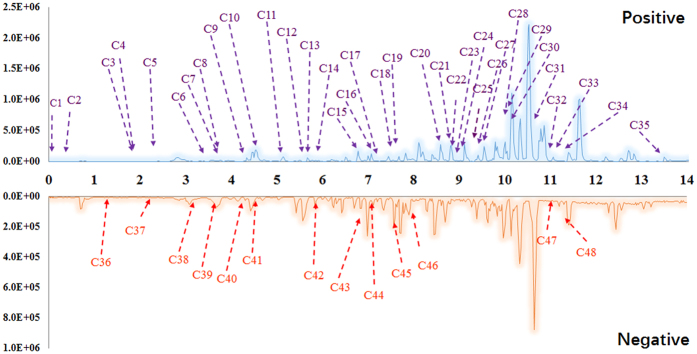
The characteristics of the base peak chromatogram (BPC) of rat serum after oral administration of AS1350 with PeakView™ in positive ion mode (POS) and negative ion mode (NEG). The information was listed in Table S2.

On the basis of the therapeutic effect of AS1350 on KYDS, the transitional components of AS1350 were determined by UPLC/MS with IDA. The structures of compounds were deduced based on high-accuracy [M − H]^−^ and [M + FA − H]^−^ (in negative ion mode) or [M + H]^+^, [M + Na]^+^ and [M + NH_4_]^+^ (in positive ion mode) on precursor ions whose product ions were used. The results were listed in Table S2 and Fig. 3, which showed that a total of 47 compounds (35 compounds in positive ion mode and 12 compounds in negative ion mode) were identified from the constituents of AS1350 such as betaine, catechin, 5-hydroxymethyl-2-furaldehyde, scoparone, clovene, stepharine, nordihydroguaiaretic acid, riboflavine, rutin, scopoletin, *etc*. These compounds were critical to the effect of KYDS.

### Correlation analysis between marker metabolites and absorbed constituents

**Figure 4:**
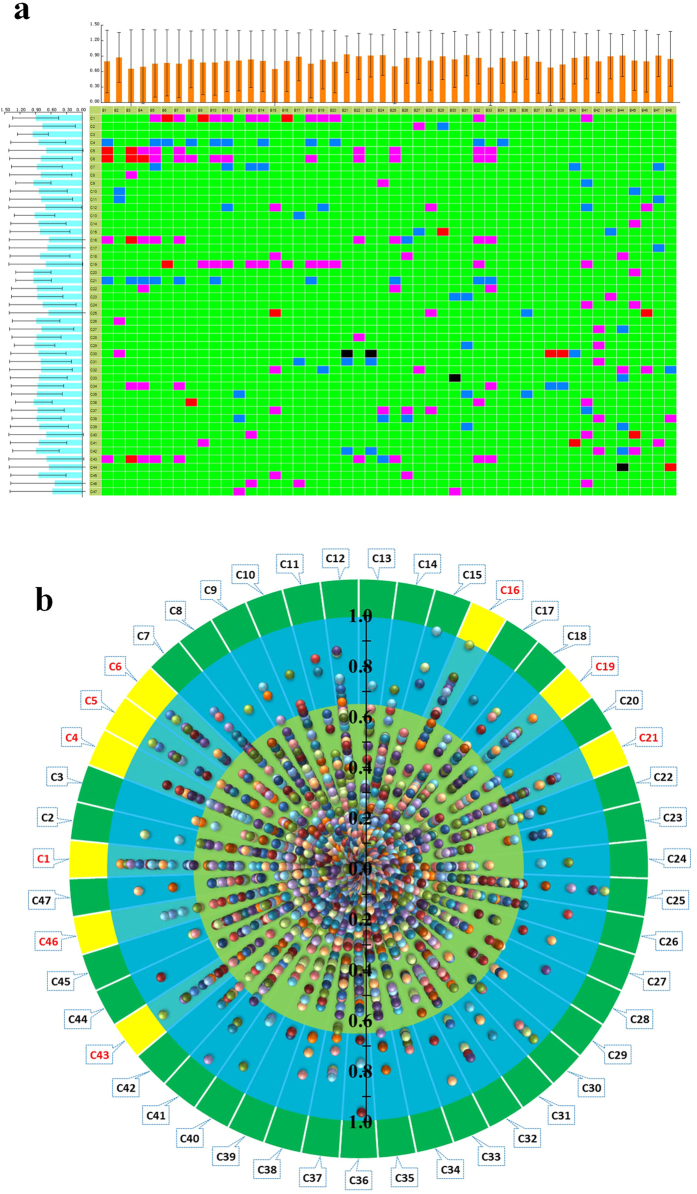
The heat-maps and scatter diagrams of correlation between marker metabolites and serum constituents in AS1350. In the heat-maps: 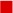, highly positively correlated; 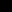, highly negatively correlated; 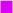, highly positively correlated; 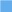, highly negatively correlated; 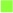, low correlation; B1 to B48 were marker metabolites (listed in Table S1); C1 to C47 were chemical components (listed in Table S2). The scatter diagram was derived from the absolute value of correlation from heat-maps, and the yellow part met the criteria: 0.65* *< |*r*|* *< 1.

Under the presupposition of therapeutic efficacy of AS1350, we extracted the correlation when its absolute value was greater than 0.65 using PCMS software to reveal which compounds contributed to the therapeutic effect of AS1350 against KYDS. According to the results shown in Fig. 4b, 9 components such as betaine, scoparone, clovene, stepharine, longipedunin C, gomisins, schizandrin, auxin A, and 1,11-undecanedicarboxylicacid were selected as highly positively, and negatively, correlated to the effects of AS1350 on KYDS.

### Potential targets prediction of the correlated constituents

**Figure 5:**
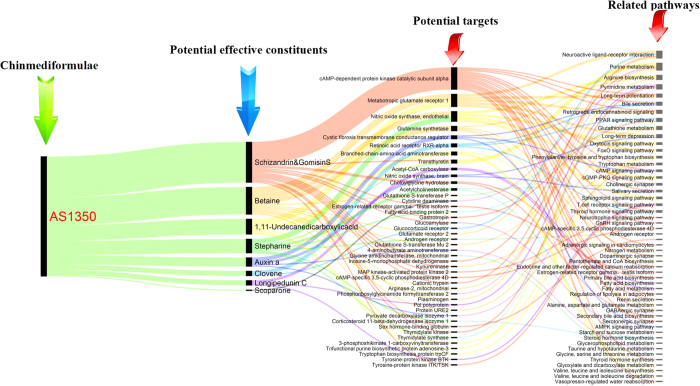
The diagram for the connection of potentially effective constituent-potential target-pathways (listed in Table S3).

After analysis by PCMS, 9 ingredients absorbed into the blood were highly correlated with the therapeutic effect of AS1350 on KYDS. To confirm the effect of these compounds, the ingredients were uploaded to the Pharmapper server (http://lilab.ecust.edu.cn/pharmmapper/index.php) to predict the protein targets, which were closely or directly related to the functional mechanisms. Prediction results discovered that 44 potential protein targets (prediction by *Z*-score ≥ 0.8^21^) were found in Table S3 and Fig. 5, including fatty acid-binding protein 2, sex hormone-binding globulin, corticosteroid 11-beta-dehydrogenase isozyme 1, androgen receptor, estrogen-related receptor gamma, testis isoform, acetylcholinesterase, cAMP-dependent protein kinase catalytic subunit alpha, cAMP-specific 3,5-cyclic phosphodiesterase 4D, tryptophan biosynthesis protein trpCF, tyrosine-protein kinase ITK/TSK, tyrosine-protein kinase BTK, choloylglycine hydrolase, metabotropic glutamate receptor 1, retinoic acid receptor RXR-alpha, nitric oxide synthase, brain *etc*. Through the KEGG pathway annotation, these potential protein targets were supposed to participate in 51 pathways, such as fatty acid biosynthesis, fatty acid metabolism, the thyroid hormone signalling pathway, thyroid hormone synthesis, androgen receptor, renin secretion, cAMP signalling pathway, cGMP-PKG signalling pathway, bile secretion, secondary bile acid biosynthesis, retrograde endocannabinoid signalling, FoxO signalling pathway, GABAergic synapse, neuroactive ligand-receptor interaction, GnRH signalling pathway, glycerophospholipid metabolism, cholinergic synapse, oestrogen-related receptor gamm, testis isoform, neurotrophin signalling pathway, primary bile acid biosynthesis, *etc*. Of concern was that the fatty acid biosynthesis, fatty acid metabolism, thyroid hormone signalling pathway, thyroid hormone synthesis, androgen receptor, renin secretion, cAMP signalling pathway, cGMP-PKG signalling pathway, and glycerophospholipid metabolism were related to the mechanisms of KYDS, which meant that schizandrin, gomisin S, betaine, scoparone, clovene, stepharine, longipedunin C, gomisins, auxin A, and 1,11-undecanedicarboxylicacid from AS1350 were active ingredients for the treatment of KYDS.

## Discussion

After the rats were induced by corticosterone over sufficient time, the hypothalamus, pituitary, and adrenal organs showed varying degrees of atrophy, especially the hypothalamus neuronal cells, which eventually led to the disorder of the hypothalamus-pituitary-adrenal axis^20^, meaning that the cellular damage or function degeneration was an important mechanism in KYDS, as found in clinical manifestations of KYDS in humans. The fatty acids were the energy source of multiple organs and tissues, whose cytomembranes were composed of phosphatide including two fatty acids^22^. Among them, n-3 polyunsaturated fatty acids (n-3 PUFAs) played a critical role in the development and the function of brain and nervous system, which was not only the necessary substance for the neurons and glial cell membrane, but also the main component of the myelin sheath, affecting the division and proliferation of nerve cells and brain cells as well as the extension of neuronal axons and the formation of new synapses^23^,^24^. Besides, α-linolenic acid was a simple constituent of n-3 PUFAs which could be turned into a type of more bioactive long-chain polyunsaturated fatty acid, such as eicosapentaenoic acid (EPA) or docosahexaenoic acid (DHA), through stearidonic acid^25^. Therefore, α-linolenic acid and stearidonic acid could directly determine the conversion process of EPA, influencing the protection of related nerve cells and brain cells. Otherwise, the biological derivative compound of fatty acids also has physiological activity, such as acetylcarnitine, a potential biomarker modulating the activity of brain neurotransmitters, the effect of which was analogous to acetylcholine, which could affect the biosynthesis of glutamate^26^,^27^. In addition, acetylcarnitine conferred a neuroprotective role on ischemic brain damage and could improve the neurological symptoms, reduce the free radical-mediated protein oxidation, and restore the brain energy metabolites^28^,^29^,^30^,^31^. Nervous system diseases such as Alzheimer’s disease, depression, diabetes, and chronic fatigue syndrome were closely related to the reduction of acetylcarnitine^32^,^33^,^34^. Therefore, long-term acetylcarnitine replacement therapy has been used for the treatment of these nervous system diseases^35^,^36^,^37^,^38^,^39^,^40^,^41^. The reduced levels of fatty acids in KYDS rats (Supplementary Figure 1a) suggested that the recovery of cells requires a lot of acetylcarnitine, α-linolenic acid, and other fatty acids. When the supply of the fatty acids could not meet the imposed, long-term, neuron demand, the burden of mitochondria biosynthesis fatty acids would be aggravated, which finally resulted in mitochondrial injury and fatty acid metabolism abnormalities. AS1350 could improve the neuroendocrine system, recover atrophic nerve cells, adrenal cells, and thyroid gland cells as evinced by its having regulated fatty acid metabolism, such as the elevated levels of α-linolenic, stearidonic acid, acetylcarnitine, and other fatty acids in serum (Supplementary Figure 1b).

In corticosterone-induced KYDS, steroid hormone biosynthesis pathway was proved to be significant, whereas the decrease in levels of hormones in the neuroendocrine system, and 3α,12β-dihydroxy-5β-cholanoic acid in serum had been verified (Supplementary Figure 1a). When exogenous corticosterone was given to rats, long-term and *in vitro*, the secretion of CRH in the hypothalamus was in negative feedback regulatory mode, inhibiting the HPA axis by disturbing the steroid hormone biosynthesis pathway^6^. Once exogenous corticosterone was removed, the inhibited nervous cells could barely regulate the lack of hormones in the HPA axis, leading to rapid onset of disorders of the entire nervous endocrine system. AS1350 could partly restore the steroid hormone biosynthesis pathway as evinced by the increasing level of hormones in the neuroendocrine system and 3α, 12β-dihydroxy-5β-cholanoic acid in serum.

Glycerolphospholipid, a main constituent composing cell membranes and organelles of biofilm, played a critical role in biological function. Glycerophosphorylcholine (GPC) was a derivative choline metabolite in the cytoplasm disintegrated from phosphatidylcholine (PC). As one of the organic penetrative cell factors, GPC could change the concentration of intracellular osmotic regulation substances and the intensity of extracellular penetration during adaptation to counteract the effects of urease and other macromolecules. In normal physiological conditions, the kidney could regulate the extracellular solute concentration by accumulating GPC, inositol, trimethyl glycine, and free amino acids from other organs in response to high osmotic pressure^42^,^43^. The methods of intracellular accumulation of osmotic substances include: increasing inositol absorption and glycerolphosphate biosynthesis, reducing GPC decomposition, and reducing the release of osmotic substances. Whether GPC was accumulated or reduced, it could identify the state of the disease. It was reported that cerebrospinal choline metabolite was increased in the on-going neurodegenerative process in Alzheimer’s patients, resulting in increased GPC levels and cytomembrane degradation, thus, GPC could be deemed as a level marker in the diagnosis of Alzheimer’s disease^44^. In our research, under the interference of exogenous corticosterone, GPC in serum of KYDS rats was increased significantly, showing the glycerolphospholipid metabolism as abnormal, improving the process of glycerophospholipid transfer into GPC, leading the degradation of cell membrane, and expressing the vacuoles of hypothalamus and the atrophy of adrenal cortex cells. Therefore, GPC can be regarded as a key biomarker to evaluate the KYDS rat model. AS1350 could partly restore the pathway, as evinced by reversing the GPC level to normal, it showed that AS1350 could regulate the extracellular solute concentration, and restrict the process of glycerophospholipid transfer into GPC.

Sphingolipids, a component of mammalian cytomembrane containing a common skeleton of sphingoid base, was made up of long silk amino acid and fatty chain acylcoenzyme A, and then converted into ceramide, sphingomyelin, glycosphingolipid, and other compounds. Besides, as part of the cell signalling process^45^, sphingolipids could be activated by G-protein-coupled-receptor or a nuclear receptor. A recent study reported that many sphingolipid constituents are parts of signal molecules, or the secondary messenger systems^46^. In normal cells, steady sphingolipid metabolism regulated basic functions such as: membrane balance, endocytosis, cell movement, nutrition transportation, protein synthesis, *etc*.^47^, however, the synthesis and degradation of sphingolipids will be disrupted when the cells were stimulated by external substance such as cytokines, hormones, X-ray or ultraviolet (UV) radiation, whose consequence was that the ceramide content increased^48^,^49^. Sphingolipid metabolites, especially sphingosine-1-phosphate(S1P), a sheath lipid derived from amide^50^, were closely related to calcium regulation^51^, cell growth, and apoptosis. Currently, S1P is the key substance in various physiological and pathophysiological processes including kidney diseases^52^, immunomodulatory disorders^53^, arteriosclerosis^54^, osteoporosis^55^, Alzheimer’s disease^56^, *etc*., the mechanism of which is associated with the action of S1P and S1P receptors^57^,^58^. Therefore, as a transmission component of cell signals, the level of S1P will indicate an increasing, or decreasing, trend on account of the influencing factors acting thereon. In our research, the KYDS rat model was copied successfully by injecting corticosterone subcutaneously to restrain the neuroendocrine system, and the results demonstrated that the S1P content in blood were increased significantly, which agreed with published findings^59^ in which glucocorticoid could activate the sphingolipid metabolism enzyme to increase S1P level in order to protect the kidney. It was discovered, in the adrenal pathological results, that many adrenal cortex cells were atrophied, perhaps because of reasons related to the disordered sphingolipid metabolism. S1P was accumulated in serum due to the exogenous corticosterone, however, S1P could not reach the level needed for activing the signal to repair damaged cells, or S1P receptor activity might be reduced. AS1350 could partly enhance the sphingolipid synthesis pathway as evinced by the increasing S1P level, which indicated that AS1350 could active cell signalling by promoting S1P synthesis to enhance its ability to undergo cytothesis.

In summary, this study investigated the influence of chemical components *in vivo* to metabolic markers with chinmedomics technology for revealing the effective substances of TCM. In accordance with pathological and biochemical characterisation of a syndrome, we established a rat model of KYDS, confirmed the overall therapeutic effect of chinmedic formulae and the corresponding relationship between KYDS and formulae. Eventually, the potential targets of the correlated constituents were verified to make sure that these potential effective substances were related to the functional mechanism of the applied formulae. The results showed that 48 biomarkers associated with fatty acid metabolism, lipid metabolism, steroid hormone biosynthesis, and amino acid metabolism were identified and AS1350 were investigated to retrace the related metabolic pathways of KYDS. The effective substances of AS1350 including: betaine, scoparone, clovene, stepharine, longipedunin C, gomisin S, schizandrin, auxin A, and 1,11-undecanedicarboxylicacid, were extracted by PCMS analysis method, whose potential protein targets participated in fatty acid biosynthesis, fatty acid metabolism, the thyroid hormone signalling pathway, thyroid hormone synthesis, androgen receptor, renin secretion, cAMP signalling pathway, cGMP-PKG signalling pathway, retrograde endocannabinoid signalling, GABAergic synapse, neuroactive ligand-receptor interaction, GnRH signalling pathway, glycerophospholipid metabolism, cholinergic synapse, oestrogen-related receptor gamma, testis isoform, neurotrophin signalling pathway, *etc*. These pathways were related to the functional mechanism of AS1350. The activity of these compounds, and related pathways, needs to be verified by subsequent assay. The chinmedomics strategy could offer a high-throughput manner of discovery and screening of potential effective constituents from TCM.

## Methods

### Chemicals and materials

Acetonitrile (HPLC grade) was obtained from Merck (Darmstadt, Germany); methanol (HPLC grade) was supplied by Fisher Scientific Corporation (Loughborough, UK); ultrapure water was purchased from Watson’s Food & Beverage Co., Ltd (Guangzhou, China). AS1350 was provided by Infinitus (China) Company Ltd (Guangzhou, China); Corticosterone was produced from Sigma Ltd. The Corticotropin releasing hormone (CRH) kit, corticosterone (CORT) kit, adrenocorticotropic hormone (ACTH) kit were provided by Beijing Huaying Biological Ltd; the triiodothyronine (T3) kit, thyroxine (T4) kit, testosterone (T) kit, 17-hydroxy-cortico-steroid (17-OHCS) kit, cyclic adenosine monophosphate (cAMP) kit, and cyclic guanosine monophosphate (cGMP) kit were provided by Nanjing Jiancheng Bioengineering Institute.

### Preparation of AS1350

AS1350 was extracted by hot water from *Cornua Cervi Pantotrichum (Lu-Rong), Cinnamomi Cortex (Rou-Gui), Radix Rehmanniae Praeparata (Shu-Di-Huang), Schisandra Chinensis Fructus (Wu-Wei-Zi), Barbary Wolfberry Fruit (Gou-Qi-Zi), Semen Juglandis (He-Tao-Ren), Arillus Longan (Long-Yan-Rou*), and *Fructus Ziziphi Jujubae (DA-Zao)* in the proportions: 2:2:10:3:10:9:12:12. After concentration, the extraction contained 1620 mg/ml of AS1350 and was considered to be a high-dose solution, then the high-dose solution was diluted to 810 mg/ml and 405 mg/ml as a moderate-dose solution and low-dose solution, respectively.

### Animals and treatments

A total of 56 male Wistar rats (mass 250 ± 10 g) were provided by Weitong-Lihua Experimental Animal Centre (Beijing, China). Rats had free access to food and drinking water under the following standard laboratory conditions: humidity of 50 ± 5%, a temperature of 25 ± 1 °C, and a 12 h light-dark cycle. After a further week of acclimatisation (individually, in metabolism cages), all the rats were randomly divided into seven groups of eight rats each: control groups 1 and 2, model groups 1 and 2, treatment groups at high dose (ASH), moderate dose (ASM), and low dose (ASL). From day 1 to day 21, rats from model groups and treatment groups were injected subcutaneously with corticosterone (10 mg/ml, dissolved in olive oil) daily at a dose of 1 ml/kg, meantime, rats in control groups were injected subcutaneously with olive oil daily at a dose of 1 ml/kg instead. On day 22, rats from control group 1 and model group 1 were sacrificed to collect biosamples for KYDS model evaluation and biomarker assay. From day 23 to day 43, rats of the treatment groups were orally administered with the corresponding concentration of AS1350 (dissolved in saline) at a dose of 1 ml/100 g once daily, at the same time; rats in control group 2 and model group 2 were orally administered saline instead. The experimental protocols were approved by the Animal Care and Use Committee of Heilongjiang University of Chinese Medicine (HUCM-2014-08717) and conducted according to the principles expressed in the Declaration of Helsinki.

### Collection of serum and tissue samples

On day 22, after the rats in three treatment groups were treated for 15 min, 1 ml blood was collected from the caudal vein for transitional component analysis. On day 43, blood was collected from the abdominal aorta, in all rats, for metabolomic analysis and clinical chemistry analysis: the blood was then transferred into tubes immediately and centrifuged at 3000 rpm for 10 min at 4 °C within 2 h of collection. Before all rats were sacrificed, the hypothalamus, pituitary, adrenal, thyroid, and testis were removed and fixed in 10% formalin for histopathological observation. All samples were collected and stored at −80 °C having been flash-frozen until analysis.

### Pre-treatment of serum sample for metabolomics

Before UPLC-MS analysis, 200 μl of thawed serum was transferred into a 1 ml centrifuge tube and mixed with 800 μl of methanol, the mixture was vortexed 1 min for protein precipitation and then it was centrifuged at 13,000 rpm for 10 min at 4 °C. Some 850 μl of supernatant was dried under nitrogen and dissolved in 200 μl of 80% methanol. All the pre-treated serum was filtered through a 0.22 μm membrane filter.

### Pre-treatment of serum components in AS1350

AS1350 and the serum samples were pre-treated as described above: 4 μl phosphoric acid was added to a 200 ul sample and then vortexed for 60 s. The mixed solution was applied to a pre-actrbate Oasis HLB solid phase extraction column (Waters, USA) which was washed with 1 ml of methanol and 1 ml of water. Then, after 1 ml of 100% water was washed through, the mixed solution was eluted by 100% methanol and the elute collected and dried under a stream of nitrogen gas at 45 °C. Each dried sample was thawed in 100 μl of 80% methanol and centrifuged at 13,000 rpm for 10 min at 4 °C, and then filtered through a 0.22 μm membrane before UPLC/MS analysis.

### Clinical chemistry analysis and histopathology analysis

Biochemical parameters of the serum were analysed on a microplate reader or an automatic counting instrument. The hypothalamic, pituitary, adrenal, and thyroid sections were stained with hematoxylin and eosin (H&E) and then observed by visible light microscopy and photographed. Image analysis was performed using Motic Medical 6.0 software (Xiamen Motic Software Engineering Co., Ltd). The histopathology analysis was done by the affiliated hospital of Heilongjiang University of Chinese Medicine.

### Chromatography conditions

Chromatographic separation was performed by UHPLC system (Eksigent Ekspert ultra LC 100, AB SCIEX, CA) consisting of a binary solvent manger, a sample manager and a column compartment. Chromatographic separation was undertaken on an HSS C_18_ column (100 mm × 2.1 mm i.d., 1.8 μm, Waters Corporation, Milford, USA). The column temperature was maintained at 40 °C. The flow rate was 0.4 ml/min. The injected sample volume was 3 μl for each run. All the samples were kept at 4 °C during the analysis. The optimal mobile phase containing a linear gradient elution program of 0.1% formic acid in water (solvent A) and 0.1% formic acid in acetonitrile (solvent B) was performed as follows: 0 min at 99% A; 0−5.5 min at 99–62% A; 5.5–7.5 min at 62–50% A; 7.5–9.0 min at 50–34% A; 9.0–13.0 min at 34–100% A; 13.0–15.0 min at 0–0% A.

### Mass spectrometry

High-definition mass spectrometry was performed on a triple TOF 5600^+^ MS/MS system (AB SCIEX, CA) equipped with an electrospray ion (ESI) source in both positive, and negative, ion mode. The optimal conditions of analysis were as follows: parameters in positive mode were an ion spray voltage of 5500 V, ion source heater temperature set to 600 °C, curtain gas pressure 35 psi, ion source gas 1 & 2 pressure 55 psi; parameters in negative mode were an ion spray voltage of 4000 V, ion source heater temperature set to 600 °C, curtain gas pressure 35 psi, and ion source gas 1 & 2 pressures of 65 psi. Meantime, to complete the TOFMS-IDA-MS/MS analysis, TOFMS and MS/MS experiments were run with 250 ms, and 100 ms, accumulation times, respectively: and the parameters of collision energy (CE) and collision energy spread (CES) were set at 35 eV and 15 eV in these MS/MS experiments. In addition, information-dependent acquisition (IDA) was used to trigger the acquisition of MS/MS spectra for ions matching the IDA criteria. DBS (dynamic background subtraction) was used to fulfil the IDA criteria. The application of DBS could intelligently differentiate the background and matrix-related MS/MS ions from the MS/MS ions of endogenous or exogenous components. Based on this, the top eight ions of endogenous, or exogenous, components in every 100-ms accumulation time interval were specific ions that matched the IDA criteria.

### Data processing and multivariate data analysis

The primary data files (formatted as wiff and wiff.scan files) for serum at days 22, and 43, were uploaded onto Progenesis QI 1.0 software (Nonlinear Dynamics, 2014, version: 1.0) respectively. The chromatographic alignment (AS1350 samples data was selected as a reference), data normalisation, and peak picking were performed by Progenesis QI for quantitative metabolomics. A three-dimensional matrix was created and then exported into EZinfo 2.0 software for multivariate data analysis. A Pareto scaling transformation was applied to the data before principal component analysis (PCA) and orthogonal partial least squares discriminant analysis (OPLS-DA) were performed. PCA, an unsupervised multivariate statistical approach, was used for variable reduction and separation into classes. Variables of interest were listed from VIP-plots constructed with OPLS-DA, which had significant contributions to discrimination between control group 1 and model group 1, and were considered as potential biomarkers. Then these selected ions were transferred back into Progenesis QI and marked with tag I, meanwhile, tag II was added to the ions whose *p*-value (as measured by Student’s *t*-test) were less than 0.05. Finally, the ions marked with both tags I and II were subjected to further identification to derive information including the molecular formula and a list of the relative intensities of each ion. The primary data files (formatted as wiff and wiff.scan files) of AS1350 and medicated serum were also uploaded onto Progenesis QI 1.0 software for the serum pharmaco-chemistry, [M + H]^+^, [M + Na]^+^ and [M + NH_4_]^+^ were selected for the positive mode process;[M − H]^−^ and [M + FA − H]^−^ were selected for the negative mode process, then chromatographic alignment (AS1350 samples data was selected as a reference) and peak picking were performed by Progenesis QI, and the ions which contained the mass fragments were marked with tag III and subjected to further identification.

### Identification and pathway analysis

The marked ions (tags I, II, and III) were found from the primary data files with the MS/MS information in Progenesis QI and were deduced by the fragmented patterns with the aid of high-resolution mass combined with the identification method which was designed to support identifications from serious of different databases, such as HMDB, LIPID MAPS (LIPID Metabolites And Pathways Strategy), and KEGG. Then the identified compounds lists, including compound molecular weights, compound names, compound identification scores, and fragmentation scores, were exported as. csv files. The molecular and structural formulae of the candidate compounds were retrieved by comparison and then confirmed by MS/MS scans for the characteristic ions and fragmentation patterns of the compounds. The construction, interaction, and pathway analysis of potential biomarkers were performed by MetaboAnalyst 3.0 software based on database sources including: HMDB (http://www.hmdb.org/), KEGG (http://www.genome.jp/kegg/), the Metlin database (http://metlin.scripps.edu/), and LIPID MAPS (http://dev.lipidmaps.org) to identify the related pathways. Pathway analysis was applied to determine the statistical significance of each pathway.

### PCMS analysis

The matrix of constituents in blood (AS1350) and the changes in biomarkers were imported into PCMS software for analysis. The parameters were set as follows: correlation 1 is 0.65, correlation 2 is 0.85, then the result was exported to a heat-map. In the heat-map, the ingredients whose absolute phase relationship value |*r*| were greater than 0.65 were extracted as highly correlated ingredients. The count of these highly correlated ingredients was considered as a standard to screen potentially effective constituents. Generally, it made sense that the count of these highly correlated ingredients accounted for 10% of the total amount of biomarkers.

### Statistical analysis

SPSS 18.0 for Windows was used for statistical analysis of the biochemical and metabolomics data. Statistically significant differences (*P *< 0.05) in mean values were calculated by Student’s *t*-test or 1-way ANOVA and Bonferroni multiple comparisons which were post-tested as appropriate. The prediction module in the EZinfo 2.0 software was used to reveal the detailed therapeutic effect on each rat in all treatment groups. PCMS software was used to calculate the correlation coefficients between compounds and biomarkers to reveal the potential effective constituents from AS1350. The pharmmapper server (http://lilab.ecust.edu.cn/pharmmapper/index.php) was used to predict the potential targets of compounds.

## Additional Information

**How to cite this article**: Liu, Q. *et al*. High-throughput chinmedomics-based prediction of effective components and targets from herbal medicine AS1350. *Sci. Rep.* **6**, 38437; doi: 10.1038/srep38437 (2016).

**Publisher's note:** Springer Nature remains neutral with regard to jurisdictional claims in published maps and institutional affiliations.

## Supplementary Material

Supplementary Information
