# Supplementary material for: High-throughput chinmedomics-based prediction of effective components and targets from herbal medicine AS1350
Source: Sci Rep. 2016 Dec 2;6:38437. doi: 10.1038/srep38437 (PMC5133595; doi:10.1038/srep38437)
Supplement: Supplementary Information [file srep38437-s1.doc]

**High-throughput chinmedomics-based prediction of effective components and targets from herbal medicine AS1350**

Qi Liu^1‡^, Aihua Zhang^1‡^, Liang Wang^1^, Guangli Yan^1^, Hongwei Zhao^2^, Hui Sun^1,3^, Shiyu Zou^2^, Jinwei Han^1^, Chung Wah Ma^2^, Ling Kong^1^, Xiaohang Zhou^1^, Yang Nan^1^, Xijun Wang^1,4^*

1. Sino-America Chinmedomics Technology Cooperation Center, Chinmedomics Research Center of TCM State Administration, National TCM Key Laboratory of Serum Pharmacochemistry, Laboratory of Metabolomics, Heilongjiang University of Chinese Medicine, Heping Road 24, Harbin 150040, China.

2. Infinitus (China) Company Ltd, Guangdong Province, China

3. Department of Pharmaceutical Analysis, Heilongjiang University of Chinese Medicine, Heping Road 24, Harbin 150040, China.

4. State Key Laboratory of Quality Research in Chinese Medicine, Macau University of Science and Technology, Avenida Wai Long,Taipa, Macau

*Address correspondence to:

Prof. Xijun Wang

Sino-America Chinmedomics Technology Cooperation Center, National TCM Key Laboratory of Serum Pharmacochemistry, Chinmedomics Research Center of TCM State Administration, Laboratory of Metabolomics, Heilongjiang University of Chinese Medicine, Heping Road, Harbin, China.

Tel. & Fax +86-451-82110818

Email: xijunwangls@126.com

^‡^These authors contributed equally to this work.

**
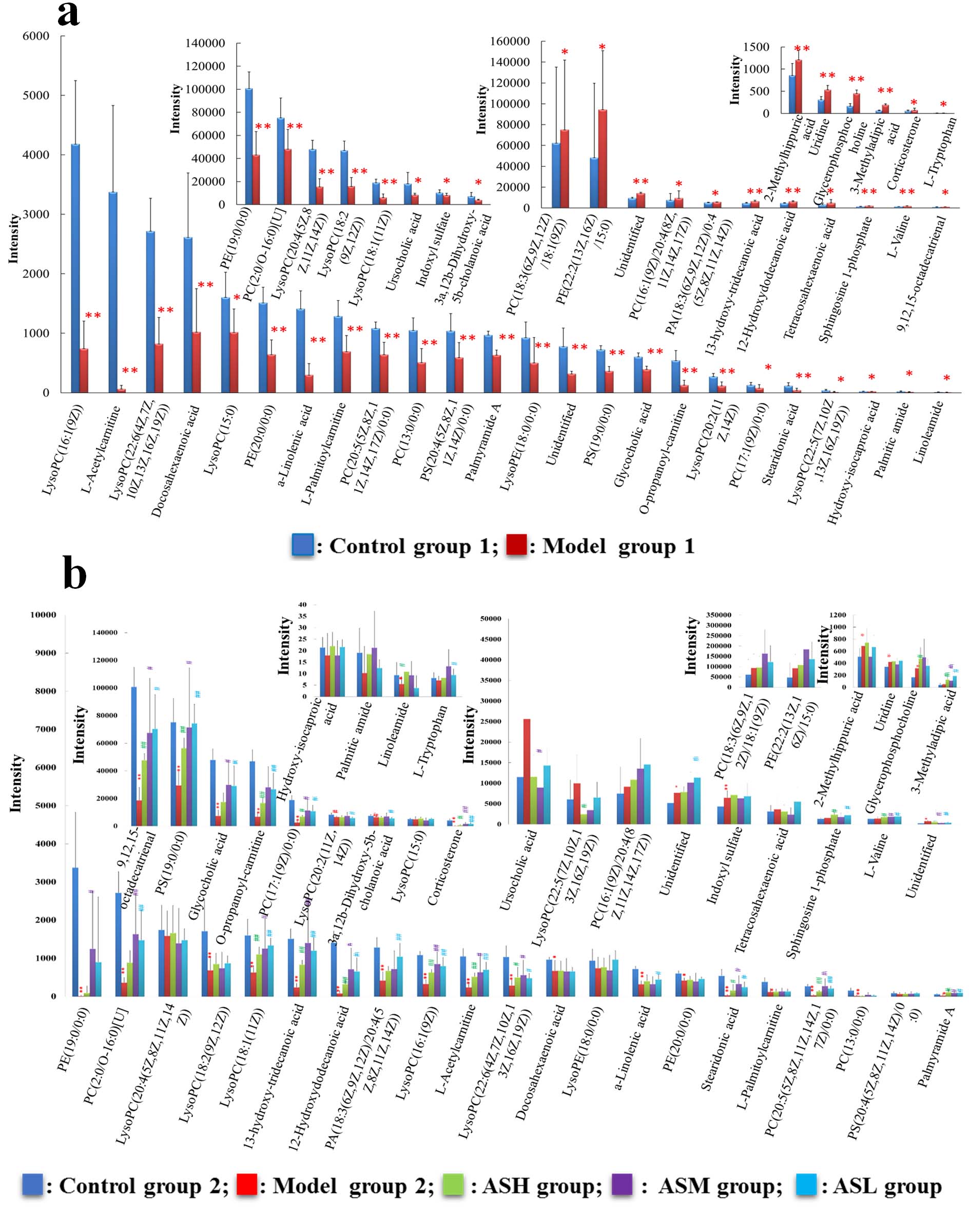
**

**Supplementary Figure 1** Relative signal intensities of biomarkers identified by TOFMS-IDA-8 MS/MS. Bar plots represent the mean relative metabolite intensity and standard deviations, error bars represent the mean±sd (In figure 3a: Student’s t-test; * significant difference from control group 1 at p<0.05, ** significant difference from control group 1 at p<0.01. In figure 3b: 1-way ANOVA with a Bonferroni correction; *significant difference from control group 2 at p<0.05/4, **significant difference from control group 2 at p<0.01/4; # significant difference from model group 2 at p<0.05/4, ## significant difference from model group 2 at p<0.01/4).The corresponding markers represented to the Supplementary Table S1.

**Table S1 Identification and pathways of potential biomarkers of KYDS based on serum metabolic profiling**

| **No.** | **Rt (min)** | **m/z determined** | **Error (ppm)** | **Ion form** | **Formula** | **Metabolite Name** | **Corresponding metabolic pathways** |
| --- | --- | --- | --- | --- | --- | --- | --- |
| B1 | 0.66 | 258.1103 | 0.8 | M+H | C_8_H_20_NO_6_P | Glycerophosphocholine | Glycerophospholipid metabolism |
| B2 | 0.68 | 118.0862 | 0.5 | M+H | C_5_H_11_NO_2_ | L-Valine | Valine, leucine and isoleucine degradation & biosynthesis |
| B3 | 1.26 | 204.1229 | 0.6 | M+H | C_9_H_17_NO_4_ | L-Acetylcarnitine | Insulin resistance pathway |
| B4 | 10.19 | 568.3372 | 4.5 | M+H | C_30_H_50_NO_7_P | LysoPC(22:6(4Z,7Z,10Z,13Z,16Z,19Z)) | Phospholipid metabolism |
| B5 | 10.21 | 520.3399 | 0.2 | M+H | C_26_H_50_NO_7_P | LysoPC(18:2(9Z,12Z)) | Glycerophospholipid metabolism |
| B6 | 10.21 | 542.3228 | 2.4 | M+H | C_28_H_48_NO_7_P | PC(20:5(5Z,8Z,11Z,14Z,17Z)/0:0) | Phospholipid metabolism |
| B7 | 10.21 | 544.3404 | 1.2 | M+H | C_28_H_50_NO_7_P | LysoPC(20:4(5Z,8Z,11Z,14Z)) | Phospholipid metabolism |
| B8 | 10.32 | 400.3444 | 4.7 | M+H | C_23_H_45_NO_4_ | L-Palmitoylcarnitine | Phospholipid metabolism |
| B9 | 9.44 | 277.2179 | 4.1 | M+H | C_18_H_28_O_2_ | Stearidonic acid | alpha-Linolenic acid metabolism |
| B10 | 10.57 | 496.3402 | 0.9 | M+H | C_24_H_50_NO_7_P | PE(19:0/0:0) | Phospholipid metabolism |
| B11 | 10.83 | 522.3556 | 0.3 | M+H | C_26_H_52_NO_7_P | LysoPC(18:1(11Z)) | Phospholipid metabolism |
| B12 | 11.02 | 546.2831 | 0.8 | M+H | C_26_H_44_NO_9_P | PS(20:4(5Z,8Z,11Z,14Z)/0:0) | Phospholipid metabolism |
| B13 | 11.09 | 510.3549 | 1.1 | M+H | C_25_H_52_NO_7_P | PE(20:0/0:0) | Phospholipid metabolism |
| B14 | 11.13 | 548.3649 | 4.3 | M+H | C_28_H_54_NO_7_P | LysoPC(20:2(11Z,14Z)) | Phospholipid metabolism |
| B15 | 11.21 | 280.2633 | 0.7 | M+H | C_18_H_33_NO | Linoleamide | Fatty acid metabolism |
| B16 | 10.34 | 279.2345 | 4.3 | M+H | C_18_H_30_O_2_ | a-Linolenic acid | alpha-Linolenic acid metabolism |
| B17 | 11.68 | 524.372 | 1.8 | M+H | C_26_H_54_NO_7_P | PC(2:0/O-16:0)[U] | Phospholipid metabolism |
| B18 | 11.91 | 562.3089 | 4.7 | M+Na | C_25_H_50_NO_9_P | PS(19:0/0:0) | Phospholipid metabolism |
| B19 | 11.95 | 466.2894 | 4.8 | M+H | C_26_H_43_NO_6_ | Glycocholic acid | Bile acid metabolism |
| B20 | 12.84 | 672.387 | 2.3 | M+H | C_36_H_53_N_3_O_9_ | Palmyramide A | Fatty acid metabolism |
| B21 | 12.95 | 780.5537 | 0.1 | M+H | C_44_H_78_NO_8_P | PC(16:1(9Z)/20:4(8Z，11Z，14Z，17Z)) | Phospholipid metabolism |
| B22 | 12.97 | 263.2373 | 1.4 | M+H | C_18_H_30_O | 9,12,15-octadecatrienal | alpha-Linolenic acid metabolism |
| B23 | 12.97 | 758.5698 | 0.5 | M+H | C_42_H_80_NO_8_P | PE(22:2(13Z,16Z)/15:0) | Phospholipid metabolism |
| B24 | 13.49 | 782.5693 | 0.2 | M+H | C_44_H_80_NO_8_P | PC(18:3(6Z,9Z,12Z)/18:1(9Z)) | Phospholipid metabolism |
| B25 | 2.02 | 218.1378 | 4.0 | M+H | C_10_H_19_NO_4_ | O-propanoyl-carnitine | Fatty acid metabolism |
| B26 | 2.42 | 205.09 | 4.8 | M+H | C_11_H_12_N_2_O_2_ | L-Tryptophan | Tryptophan metabolism |
| B27 | 5.87 | 133.0863 | 2.9 | M+H | C_6_H_12_O_3_ | Hydroxyisocaproic acid | Leucine metabolism |
| B28 | 7.01 | 736.4988 | 4.4 | M+NH4 | C_41_H_67_O_8_P | PA(18:3(6Z,9Z,12Z)/20:4(5Z,8Z,11Z,14Z)) | Phospholipid metabolism |
| B29 | 7.08 | 347.2228 | 3.2 | M+H | C_21_H_30_O_4_ | Corticosterone | Steroid hormone biosynthesis |
| B30 | 8.22 | 256.2625 | 3.9 | M+H | C_16_H_33_NO | Palmitic amide | Fatty acid metabolism |
| B31 | 8.88 | 357.2797 | 2.5 | M+H | C_24_H_36_O_2_ | Tetracosahexaenoic acid | alpha-Linolenic acid metabolism |
| B32 | 9.58 | 380.2566 | 1.5 | M+H | C_18_H_38_NO_5_P | Sphingosine 1-phosphate | [Sphingolipid metabolism](http://www.metaboanalyst.ca/faces/Secure/pathway/ResultView.xhtml) |
| B33 | 9.87 | 494.3234 | 1.5 | M+H | C_24_H_48_NO_7_P | LysoPC(16:1(9Z)) | Phospholipid metabolism |
| B34 | 6.42 | 157.1245 | - | - | - | Unidentified | - |
| B35 | 3.75 | 159.0671 | 4.9 | M-H | C_7_H_12_O_4_ | 3-Methyladipic acid | Fatty acid metabolism |
| B36 | 4.41 | 192.0671 | 2.5 | M-H | C_10_H_11_NO_3_ | 2-Methylhippuric acid | Fatty acid metabolism |
| B37 | 0.7 | 215.0328 | - | - | - | Unidentified | - |
| B38 | 8.96 | 215.1654 | 0.6 | M-H | C_12_H_24_O_3_ | 12-Hydroxydodecanoic acid | Fatty acid metabolism |
| B39 | 9.69 | 229.181 | 0.3 | M-H | C_13_H_26_O_3_ | 13-hydroxy-tridecanoic acid | Fatty acid metabolism |
| B40 | 1.03 | 243.0626 | 1.4 | M-H | C_9_H_12_N_2_O_6_ | Uridine | Pyrimidine metabolism |
| B41 | 12.74 | 327.2325 | 1.4 | M-H | C_22_H_32_O_2_ | Docosahexaenoic acid | Fatty acid metabolism |
| B42 | 10.75 | 614.345 | 0.4 | M+FA-H | C_30_H_52_NO_7_P | LysoPC(22:5(7Z,10Z,13Z,16Z,19Z)) | Phospholipid metabolism |
| B43 | 10.91 | 506.3234 | 3.6 | M-H | C_25_H_50_NO_7_P | PC(17:1(9Z)/0:0) | Phospholipid metabolism |
| B44 | 9.93 | 437.2893 | 1.1 | M+FA-H | C_24_H_40_O_4_ | 3a,12b-Dihydroxy-5b-cholanoic acid | Steroid hormone biosynthesis |
| B45 | 8.72 | 453.2841 | 1.3 | M+FA-H | C_24_H_40_O_5_ | Ursocholic acid | Secondary bile acid biosynthesis |
| B46 | 11.59 | 482.3251 | 2.0 | M+H | C_23_H_48_NO_7_P | LysoPC(15:0) | Phospholipid metabolism |
| B47 | 11.68 | 480.3087 | 1.8 | M-H | C_23_H_48_NO_7_P | LysoPE(18:0/0:0) | Phospholipid metabolism |
| B48 | 4.13 | 212.0025 | 0.9 | M-H | C_8_H_7_NO_4_S | Indoxyl sulfate | Tryptophan metabolism |

**Table S2 Identification of the constituents in rat serum after oral administration of AS1350 oral liquid**

| **NO.** | **Rt** | **m/z determined** | **Error**  **（ppm）** | **Ion form** | **Molecular**  **Formula** | **Compouds Name** | **Origin** |
| --- | --- | --- | --- | --- | --- | --- | --- |
| C1 | 0.74 | 118.0858 | 3.90 | M+H | C_5_H_11_NO_2_ | Betaine | ⑤ |
| C2 | 0.91 | 291.0841 | 4.59 | M+H | C_15_H_14_O_6_ | Catechin | ⑥ |
| C3 | 2.24 | 127.0388 | 1.34 | M+H | C_6_H_6_O_3_ | 5-Hydroxymethyl-2-furaldehyde | ① |
| C4 | 2.24 | 224.0912 | 2.37 | M+NH_4_ | C_11_H_10_O_4_ | Scoparone | ④ |
| C5 | 2.84 | 227.1748 | 4.77 | M+Na | C_15_H_24_ | Clovene | ④ |
| C6 | 3.96 | 298.1437 | 0.23 | M+H | C_18_H_19_NO_3_ | Stepharine | ⑧ |
| C7 | 4.02 | 344.1855 | 0.41 | M+ACN+H | C_18_H_22_O_4_ | Nordihydroguaiaretic acid | ④ |
| C8 | 4.04 | 377.1457 | -0.37 | M+H | C_17_H_20_N_4_O_6_ | Riboflavine | ⑤⑧ |
| C9 | 4.87 | 611.1656 | -4.08 | M+H | C_27_H_30_O_16_ | Rutin | ⑧ |
| C10 | 4.98 | 193.0490 | 2.80 | M+H | C_10_H_8_O_4_ | Scopoletin | ⑤ |
| C11 | 5.48 | 193.1581 | 3.05 | M+H | C_13_H_20_O | β-Ionone | ③ |
| C12 | 5.90 | 453.3474 | 4.26 | M+K | C_29_H_50_O | β-Sitosterol | ⑤⑧ |
| C13 | 5.99 | 147.0435 | 3.81 | M+H | C_9_H_6_O_2_ | Coumarin | ⑤ |
| C14 | 6.17 | 194.1171 | 2.37 | M+NH_4_ | C_11_H_12_O_2_ | Cinnamyl acetate | ② |
| C15 | 7.06 | 219.1740 | 1.55 | M+H | C_15_H_22_O | Nootkatone | ④ |
| C16 | 7.48 | 507.2028 | -2.88 | M+H | C_29_H_30_O_8_ | Longipedunin C | ④ |
| C17 | 7.48 | 537.2103 | 3.00 | M+H | C_30_H_32_O_9_ | GomisinC | ④ |
| C18 | 7.75 | 373.1659 | -3.56 | M+H | C_21_H_24_O_6_ | Kadsurin A | ④ |
| C19 | 7.80 | 419.2076 | -2.79 | M+H | C_23_H_30_O_7_ | GomisinS | ④ |
| C20 | 8.86 | 401.1619 | -4.03 | M+H | C_22_H_24_O_7_ | GomisinR | ④ |
| C21 | 9.06 | 433.2231 | -2.35 | M+H | C_24_H_32_O_7_ | Schizandrin | ④ |
| C22 | 9.06 | 548.2489 | 0.22 | M+H | C_28_H_34_O_10_ | GomisinD | ④ |
| C23 | 9.28 | 417.1936 | -4.76 | M+H | C_23_H_28_O_7_ | Gomisin A | ④ |
| C24 | 9.30 | 518.2392 | -1.43 | M+NH_4_ | C_27_H_32_O_9_ | SchisantherinC | ④ |
| C25 | 9.70 | 439.1731 | -0.87 | M+Na | C_23_H_28_O_7_ | GomisinO | ④ |
| C26 | 9.81 | 401.1969 | -2.57 | M+H | C_23_H_28_O_6_ | Deoxygomisin A | ④ |
| C27 | 9.81 | 501.2504 | -4.19 | M+H_4_ | C_28_H_36_O_8_ | Angeloylgomisin H | ④ |
| C28 | 10.09 | 532.2551 | -1.86 | M+NH_4_ | C_28_H_34_O_9_ | Tigloylgomisin P | ④ |
| C29 | 10.12 | 251.2008 | -0.96 | M+H | C_16_H_26_O_2_ | Sclareolide | ⑧ |
| C30 | 10.36 | 532.2554 | -2.42 | M+H | C_28_H_34_O_9_ | Angeloylgomisin P | ④ |
| C31 | 10.74 | 515.2290 | -2.79 | M+H | C_28_H_34_O_9_ | GomisinF | ④ |
| C32 | 11.11 | 149.0224 | -4.76 | M+H | C_5_H_8_O_3_S | 2-oxo-4-methylthio-butanoic acid | ① |
| C33 | 11.29 | 417.2284 | -2.95 | M+H | C_24_H_32_O_6_ | Deoxyschizandrin | ④ |
| C34 | 11.33 | 453.3344 | 4.24 | M+H | C_30_H_44_O_3_ | Zizyberenalic acid | ⑧ |
| C35 | 13.51 | 429.4051 | 4.29 | M+H | C_30_H_52_O | Cycloartanol | ⑤ |
| C36 | 1.89 | 407.1185 | -0.14 | M+FA-H | C_15_H_22_O_10_ | Catalpol | ③ |
| C37 | 2.51 | 329.0874 | 1.10 | M-H | C_14_H_18_O_9_ | 3'-Glucosyl-2'，4'，6'-trihydroxyacetophenone | ⑧ |
| C38 | 3.61 | 523.1647 | 4.16 | M-H | C_21_H_32_O_15_ | RehmanniosideB | ③ |
| C39 | 4.03 | 375.13 | -0.80 | M-H | C_16_H_24_O_10_ | Loganate | ③ |
| C40 | 4.69 | 403.1598 | -1.75 | M-H | C_25_H_24_O_5_ | Laxifolin | ⑧ |
| C41 | 4.99 | 193.0507 | -0.16 | M-H | C_10_H_10_O_4_ | Ferulic acid | ②⑤ |
| C42 | 6.25 | 312.1236 | -1.69 | M+FA-H | C_17_H_17_NO_2_ | Asimilobine | ⑧ |
| C43 | 6.99 | 327.2177 | 3.40 | M-H | C18H32O5 | Auxin a | ② |
| C44 | 7.49 | 491.1182 | 0.46 | M+FA-H | C_22_H_22_O_10_ | Swertisin | ⑧ |
| C45 | 7.86 | 557.201 | 3.28 | M-H | C_29_H_34_O_11_ | LancifodilactoneB | ④ |
| C46 | 8.32 | 243.1604 | -0.84 | M-H | C_13_H_24_O_4_ | 1，11-Undecanedicarboxylicacid | ① |
| C47 | 11.22 | 507.2709 | 4.35 | M+FA-H | C_30_H_38_O_4_ | LancilactoneB | ④ |

①*: Cornua Cervi Pantotrichum;* ②*: Cinnamomi Cortex;* ③*: Radix Rehmanniae Praeparata;* ④*: Schisandra Chinensis Fructus;* ⑤*: Barbary Wolfberry Fruit;* ⑥*: Semen Juglandis;* ⑦*: Arillus Longan;* ⑧*: Fructus Ziziphi Jujubae.*

**Table S3 The proteins retrieved by Pharmapper tool as the best target proteins ranked by Z-score**

| **Compound name** | **Target name** | **Fit score** | **Normalized**  **fit score** | **Z-score** |
| --- | --- | --- | --- | --- |
| Betaine | Metabotropic glutamate receptor 1 | 2.92 | 0.58 | 1.66 |
|  | Glutamate receptor 2 | 2.88 | 0.58 | 1.49 |
|  | Arginase-2, mitochondrial | 2.87 | 0.41 | 1.39 |
|  | Glycine amidinotransferase, mitochondrial | 2.88 | 0.72 | 1.38 |
|  | Plasminogen | 2.85 | 0.71 | 1.37 |
|  | Glutathione S-transferase Mu 2 | 2.89 | 0.32 | 1.34 |
|  | Nitric oxide synthase, endothelial | 2.79 | 0.56 | 1.27 |
|  | Glutamine synthetase | 2.86 | 0.57 | 1.18 |
|  | 3-phosphoshikimate 1-carboxyvinyltransferase | 2.81 | 0.56 | 1.08 |
|  | Protein URE2 | 2.83 | 0.4 | 1.03 |
|  | Glutathione S-transferase P | 3.18 | 0.23 | 0.99 |
| Clovene | Retinoic acid receptor RXR-alpha | 5.73 | 0.64 | 2.49 |
|  | Glucocorticoid receptor | 5.73 | 0.72 | 1.78 |
| Stepharine | Androgen receptor | 4.59 | 0.57 | 2.37 |
|  | Acetylcholinesterase | 4.42 | 0.44 | 2.51 |
|  | Transthyretin | 4.04 | 0.67 | 1.28 |
|  | Thymidylate synthase | 3.95 | 0.99 | 1.83 |
|  | Fatty acid-binding protein 2 | 4.09 | 0.58 | 1.00 |
|  | Corticosteroid 11-beta-dehydrogenase isozyme 1 | 3.96 | 0.79 | 1.12 |
| Longipedunin C | Acetyl-CoA carboxylase | 4.31 | 0.48 | 1.46 |
|  | Pol polyprotein | 4.46 | 0.45 | 2.51 |
|  | Tyrosine-protein kinase BTK | 4.20 | 0.35 | 0.95 |
| Schizandrin& GomisinS | Choloylglycine hydrolase | 4.37 | 0.55 | 2.08 |
|  | Gastrotropin | 4.03 | 0.58 | 2.05 |
|  | Inosine-5-monophosphate dehydrogenase | 3.9 | 0.43 | 1.8 |
|  | Tyrosine-protein kinase ITK/TSK | 3.78 | 0.63 | 1.68 |
|  | cAMP-specific 3,5-cyclic phosphodiesterase 4D | 3.79 | 0.54 | 1.66 |
|  | Estrogen-related receptor gamma，testis isoform | 3.74 | 0.62 | 1.49 |
|  | MAP kinase-activated protein kinase 2 | 3.76 | 0.54 | 1.44 |
|  | Cationic trypsin | 3.79 | 0.47 | 1.33 |
|  | cAMP-dependent protein kinase catalytic subunit alpha | 3.84 | 0.35 | 1.29 |
|  | Kynureninase | 3.56 | 0.59 | 1.01 |
|  | Glucoamylase | 3.63 | 0.36 | 0.97 |
|  | Thymidylate kinase | 3.64 | 0.46 | 0.88 |
|  | Sex hormone-binding globulin | 3.87 | 0.48 | 0.83 |
| Auxin a | Pyruvate decarboxylase isozyme 1 | 4.35 | 0.44 | 1.18 |
|  | 4-aminobutyrate aminotransferase | 4.73 | 0.53 | 1.05 |
|  | Cystic fibrosis transmembrane conductance regulator | 5.59 | 0.51 | 0.82 |
|  | Nitric oxide synthase, brain | 4.04 | 0.5 | 0.76 |
| 1,11-Undecanedicarboxylicacid | Tryptophan biosynthesis protein trpCF | 4.62 | 0.58 | 2.51 |
|  | Phosphoribosylglycinamide formyltransferase 2 | 4.8 | 0.53 | 1.64 |
|  | Trifunctional purine biosynthetic protein adenosine-3 | 4.62 | 0.42 | 1.5 |
|  | Branched-chain-amino-acid aminotransferase | 4.58 | 0.35 | 1.49 |
| Scoparone | Cytidine deaminase | 3.27 | 0.65 | 0.97 |
